# Supplementary material for: False-negative results of initial RT-PCR assays for COVID-19: A systematic review
Source: PLoS One. 2020 Dec 10;15(12):e0242958. doi: 10.1371/journal.pone.0242958 (PMC7728293; doi:10.1371/journal.pone.0242958)
Supplement: S1 File — (PDF) [file pone.0242958.s001.pdf]

S1 File. PICO question/ eligibility criteria

	Include	Exclude
Population	Patients under suspicion of infection by clinical or epidemiological criteria at initial testing (first healthcare encounter, initial ED attendance)<br>• Due to clinical suspicious<br>• Due to contact tracing	• Populations assessed for discharge or at different setting to initial testing<br>• Population screening<br>• spiked or artificially contrived samples<br>• Less or equal to 10 confirmed cases<br>• Special populations: cancer patients, multiple sclerosis, pregnant women, diabetes patients, gynaecological cancer, HIV, surgical patients (pre-operative screening)
Index test	RT-PCR, including alternative methods for RNA extraction and other technologies (only if fulfil other criteria)	• Sample pooling<br>• Machine learning
Reference standard	• Any RT-PCR positive finding (in a short term)<br>• PCR plus clinical signs/symptoms<br>• Any other criteria including PCR findings	• Serological tests<br>• rapid PCR-based tests<br>• Clinical guideline diagnosis (e.g. WHO or China CDC)<br>• WHO classification criteria<br>• Consensus of experts/clinicians
Outcome	False- negative cases	Absolute numbers without a total of COVID-19 confirmed patients, insufficient/unclear numerical information
Study design	observational studies (including accuracy studies, cohorts, and case series)	• Comparative studies between types of specimens focus on concordance<br>• Comparison of alternative methods for extraction of RNA (e.g. RT-LAMP or CRISPr technology), rapid PCR-based tests, point-of-care or near patient antigen or PCR based tests<br>• Studies reporting only analytical sensitivity (i.e. limits of detection) or analytical specificity (e.g. cross-reactivity), also validation/development of novel assays<br>• Mathematical modelling<br>• Proof of concept studies
