# Supplementary material for: False-negative results of initial RT-PCR assays for COVID-19: A systematic review
Source: PLoS One. 2020 Dec 10;15(12):e0242958. doi: 10.1371/journal.pone.0242958 (PMC7728293; doi:10.1371/journal.pone.0242958)
Supplement: S2 File — (PDF) [file pone.0242958.s002.pdf]

**S2 File. SEARCH STRATEGIES**

| | |
|---|---|
| MEDLINE-PUBMED | #14 Add Search ((((\"Wuhan coronavirus\" [Supplementary Concept] OR \"COVID-19\" OR \"2019 ncov\"[tiab] OR ((\"novel coronavirus\"[tiab] OR \"new coronavirus\"[tiab]) AND (wuhan[tiab] OR 2019[tiab])) OR 2019-nCoV[All Fields] OR (wuhan[tiab] AND coronavirus[tiab])))))))) AND (((((("Polymerase Chain Reaction"[Mesh]) OR "Real-Time Polymerase Chain Reaction"[Mesh]) OR Real-Time Polymerase Chain Reaction) OR real time pcr) OR PCR [tiab]) OR RT-PCR kit*[tiab]) OR RT-PCR assay [tiab])<br>#13 Add Search (((((("Polymerase Chain Reaction"[Mesh]) OR "Real-Time Polymerase Chain Reaction"[Mesh]) OR Real-Time Polymerase Chain Reaction) OR real time pcr) OR PCR [tiab]) OR RT-PCR kit*[tiab]) OR RT-PCR assay [tiab]<br>#12 Add Search RT-PCR assay [tiab]<br>#11 Add Search RT-PCR kit*[tiab]<br>#10 Add Search PCR [tiab]<br>#8 Add Search real time pcr<br>#7 Add Search Real-Time Polymerase Chain Reaction<br>#6 Add Search "Real-Time Polymerase Chain Reaction"[Mesh]<br>#5 Add Search "Polymerase Chain Reaction"[Mesh]<br>#3 Add Search ((\"Wuhan coronavirus\" [Supplementary Concept] OR \"COVID-19\" OR \"2019 ncov\"[tiab] OR ((\"novel coronavirus\"[tiab] OR \"new coronavirus\"[tiab]) AND (wuhan[tiab] OR 2019[tiab])) OR 2019-nCoV[All Fields] OR (wuhan[tiab] AND coronavirus[tiab]))))) |
| EMBASE | 1 Wuhan coronavirus.mp.<br>2 COVID-19.ti,ab.<br>3 2019 ncov.ti,ab.<br>4 novel coronavirus.ti,ab.<br>5 new coronavirus.ti,ab.<br>6 (wuhan or "2019").ti,ab.<br>7 2019-nCoV.af.<br>8 (wuhan and coronavirus).ti,ab.<br>9 or/1-8<br>10 RNA test.mp.<br>11 Real-Time Polymerase Chain Reaction/<br>12 real time PCR.ti,ab.<br>13 nucleic acid amplification test*.ti,ab.<br>14 RT-PCR kit*.ti,ab.<br>15 rRT-PCR.ti,ab.<br>16 or/10-15<br>17 9 and 16<br>18 limit 17 to last year |
| LILACS | tw:((tw:((tw:(wuhan coronavirus)) OR (tw:(covid-19)) OR (tw:(2019 ncov)) OR (tw:(novel coronavirus)) OR (tw:(new coronavirus)) OR (tw:(wuhan OR "2019")) OR (tw:(wuhan AND coronavirus)))) AND (tw:((tw:(rna test)) OR (tw:(real-time polymerase chain reaction)) OR (tw:(real time pcr)) OR (tw:(nucleic acid amplification test*)) OR (tw:(rt-pcr kit*)) OR (tw:(rrt-pcr))))) AND ( db:("LILACS" OR "PAHOIRIS" OR "BINACIS" OR "IBECS")) |
