# Supplementary material for: False-negative results of initial RT-PCR assays for COVID-19: A systematic review
Source: PLoS One. 2020 Dec 10;15(12):e0242958. doi: 10.1371/journal.pone.0242958 (PMC7728293; doi:10.1371/journal.pone.0242958)
Supplement: S3 File — (PDF) [file pone.0242958.s003.pdf]

S3 File. CHARACTERISTIC OF EXCLUDED STUDIES

ID	Commentary
Alcoba 2020 (1)	Ineligible setting: no/unclear info about initial testing
Altamirano 2020 (2)	Ineligible setting: no/unclear info about initial testing
Apra 2020 (3)	Other reasons
Arnaout 2020 (4)	Ineligible setting: no/unclear info about initial testing
Awargal 2020 (5)	FN data cannot be extracted
Bai, Harrison X 2020 (6)	Ineligible reference standard
Blain 2020 (7)	FN data cannot be extracted
Brill 2020 (8)	FN data cannot be extracted
Butler 2020 (9)	Ineligible setting: no/unclear info about initial testing
Byrne 2020 (10)	Other reasons
Callahan 2020 (11)	Other reasons
Caruso D 2020 (12)	FN data cannot be extracted
Cassaniti IN 2020 (13)	FN data cannot be extracted
Chan JFW 2020(14)	Ineligible setting: no/unclear info about initial testing
Chang 2020 (15)	Other reasons
Chau N 2020 (16)	Ineligible setting: no/unclear info about initial testing
Chen C 2020 (17)	FN data cannot be extracted
Chen JHK 2020 (18)	Ineligible setting: no/unclear info about initial testing
Chen S 2020 (19)	Other reasons
Cheng Z 2020 (20)	Ineligible setting: no/unclear info about initial testing
Clark 2020 (21)	FN data cannot be extracted
Corral 2020 (22)	Other reasons
Craney 2020 (23)	Ineligible setting: no/unclear info about initial testing
Dai WC 2020 (24)	Case report
David A. Schwartz 2020 (25)	Other reasons
De la Iglesia 2020 (26)	Ineligible setting: no/unclear info about initial testing
De Smet 2020 (27)	FN data cannot be extracted
Debray 2020 (28)	Ineligible reference standard
Desmet 2020 (29)	Other reasons
Dharavath 2020 (30)	Ineligible setting: no/unclear info about initial testing
Fernández-Pittol 2020 (31)	Ineligible setting: no/unclear info about initial testing
Freire 2020 (32)	Ineligible setting: no/unclear info about initial testing
Freire-Paspuel 2020 (33)	Ineligible setting: no/unclear info about initial testing
Gezer 2020 (34)	Ineligible reference standard
Ghinai, Isaac 2020 (35)	Case report
Greene 2020 (36)	Ineligible setting: no/unclear info about initial testing
Guan W 2020 (37)	Ineligible setting: no/unclear info about initial testing
Guo, L 2020 (38)	Ineligible population: based on samples
Han H 2020 (39)	Case report
Hirotsu 2020 (40)	Ineligible setting: no/unclear info about initial testing
Hu 2020 (41)	Case report

Hu Z 2020 (42)	FN data cannot be extracted
Hua 2020 (43)	FN data cannot be extracted
Huang 2020a (44)	Case report
Huang 2020b (45)	No info about initial RT-PCR
Ikeda 2020 (46)	FN data cannot be extracted
Israeli 2020 (47)	Ineligible setting: no/unclear info about initial testing
J.C.L. Rodrigues 2020 (48)	Other reasons
Jeffrey P. Kanne, 2020 (49)	Other reasons
Jing-Wen Ai 2020 (50)	Case report
Kinloch 2020 (51)	Ineligible setting: no/unclear info about initial testing
Lagier JC 2020 (52)	FN data cannot be extracted
Leber 2020 (53)	Other reasons
LeBlanc 2020 (54)	Ineligible setting: no/unclear info about initial testing
Leung 2020 (55)	Ineligible setting: no/unclear info about initial testing
Li D 2020 (56)	Case report
Li Y 2020 (57)	CT Findings only
Li Z 2020 (58)	No info about initial RT-PCR
Lieberman 2020 (59)	Ineligible setting: no/unclear info about initial testing
Lin L (60)	Ineligible setting: no/unclear info about initial testing
Ling Z (61)	Case report
Liu H 2020 (62)	FN data cannot be extracted
Liu R 2020 (63)	Ineligible setting: no/unclear info about initial testing
Liu, Kai-Cai 2020 (64)	Ineligible setting: no/unclear info about initial testing
Long Q 2020 (65)	Other reasons
Lu R 2020 (66)	FN data cannot be extracted
Luo, Si-Hui 2020 (67)	Case report
Matzkies 2020 (68)	Ineligible setting: no/unclear info about initial testing
Medetalibeyoğlu 2020 (69)	FN data cannot be extracted
Mesoraca 2020 (70)	Ineligible setting: no/unclear info about initial testing
Miao C 2020 (71)	Ineligible setting: no/unclear info about initial testing
Midgley CM 2020 (72)	Ineligible population: based on samples
Minjin Wang 2020 (73)	Other reasons
Miranda 2020 (74)	FN data cannot be extracted
Moore 2020 (75)	Ineligible setting: no/unclear info about initial testing
Muenchhoff 2020 (76)	Ineligible setting: no/unclear info about initial testing
Murat 2020 (77)	FN data cannot be extracted
Norooznezhad 2020 (78)	FN data cannot be extracted
Okamaoto 2020 (79)	Ineligible setting: no/unclear info about initial testing
Okba N 2020 (80)	Ineligible population: based on samples
Pan YL 2020 (81)	FN data cannot be extracted
Paramita 2020 (82)	FN data cannot be extracted
Park 2020 (83)	FN data cannot be extracted
Patel 2020 (84)	FN data cannot be extracted

Patel 2020b (85)	FN data cannot be extracted
Petrovan 2020 (86)	Ineligible population: based on samples
Pezzi 2020 (87)	Ineligible setting: no/unclear info about initial testing
Pham 2020 (88)	Ineligible setting: no/unclear info about initial testing
Poggiali E 2020 (89)	Ineligible setting: no/unclear info about initial testing
Qiu, Y 2020 (90)	FN data cannot be extracted
Reijns 2020 (91)	Ineligible setting: no/unclear info about initial testing
Ren X 2020 (92)	Unclear confirmation of cases/methodology
Salehi S 2020 (93)	Other reasons
Shirato 2020 (94)	Other reasons
Thomas-Rüddel, D 2020 (95)	Other reasons
Steuwe 2020 (96)	FN data cannot be extracted
Szymczak 2020 (97)	Ineligible setting: no/unclear info about initial testing
Takeuchi 2020 (98)	Other reasons
THIEUX 2020 (99)	Inelegible reference standard
To 2020 (100)	le setting: no/unclear info about initial testing
Tung-Chen 2020 (101)	FN data cannot be extracted
Valent 2020 (102)	FN data cannot be extracted
van Kasteren 2020 (103)	Ineligible setting: no/unclear info about initial testing
Vancheri 2020 (104)	Other reasons
Velu 2020 (105)	FN data cannot be extracted
Visseaux 2020 (106)	Ineligible setting: no/unclear info about initial testing
Vlek 2020 (107)	Other reasons
Wang 2020 (108)	Ineligible setting: no/unclear info about initial testing
Wang M 2020 (109)	Ineligible setting: no/unclear info about initial testing
Wang X 2020 (110)	Ineligible setting: no/unclear info about initial testing
Wang W 2020 (111)	Ineligible population: based on samples
Wang B 2020 (112)	Ineligible setting: no/unclear info about initial testing
Wei M 2020 (113)	Ineligible setting: no/unclear info about initial testing
Wirden 2020 (114)	Ineligible setting: no/unclear info about initial testing
Won, Joungha 2020 (115)	Other reasons
Wu J 2020 (116)	Ineligible setting: no/unclear info about initial testing
Wu J 2020b (117)	Ineligible setting: no/unclear info about initial testing
Xiong Z 2020 (118)	No info about initial RT-PCR
Xiao AT 2020 (119)	No info about initial RT-PCR
XIAO 2020b (120)	Ineligible setting: no/unclear info about initial testing
Xiao 2020c (121)	FN data cannot be extracted
Xie C 2020 (122)	Ineligible population: based on samples
Xiong Y 2020 (123)	Ineligible setting: no/unclear info about initial testing
Xu T 2020 (124)	Ineligible setting: no/unclear info about initial testing
Xu YH 2020 (125)	Ineligible setting: no/unclear info about initial testing
Yan Y 2020 (126)	Ineligible population: based on samples
Ye, Zheng 2020 (127)	Ineligible reference standard

Yoon SH 2020 (128)	Ineligible setting: no/unclear info about initial testing
Yu FY 2020 (129)	Ineligible setting: no/unclear info about initial testing
Yuanyuan L 2020 (130)	Unclear confirmation of cases/methodology
Zeng Z 2020 (131)	FN data cannot be extracted
Zhang JC 2020 (132)	Unclear confirmation of cases/methodology
Zhao J 2020 (133)	Unclear confirmation of cases/methodology
Zhao, Dahai 2020 (134)	No info about initial RT-PCR
Zhen 2020 (135)	Ineligible setting: no/unclear info about initial testing
Zheng 2020 (136)	FN data cannot be extracted
Zhongliang Wang 2020 (137)	No info about initial RT-PCR

REFERENCES

1. Alcoba-Florez J, Gil-Campesino H, Garcia-Martinez de Artola D, Gonzalez-Montelongo R, Valenzuela-Fernandez A, Ciuffreda L, et al. Sensitivity of different RT-qPCR solutions for SARS-CoV-2 detection. medRxiv. 2020.
2. Altamirano J, Govindarajan P, Blomkalns AL, Kushner LE, Stevens BA, Pinsky BA, et al. Assessment of Sensitivity and Specificity of Patient-Collected Lower Nasal Specimens for Sudden Acute Respiratory Syndrome Coronavirus 2 Testing. JAMA Netw Open. 2020;3(6):e2012005.
3. Apra C, Caucheteux C, Mensch A, Mansour J, Bernaux M, dechartres A, et al. Predictive usefulness of PCR testing in different patterns of Covid-19 symptomatology - Analysis of a French cohort of 12,810 outpatients. medRxiv. 2020:2020.06.07.20124438.
4. Arnaout R, Lee RA, Lee GR, Callahan C, Yen CF, Smith KP, et al. SARS-CoV2 Testing: The Limit of Detection Matters. bioRxiv. 2020.
5. Agarwal V, Venkatakrishnan AJ, Puranik A, Kirkup C, Lopez-Marquez A, Challener DW, et al. Long-term SARS-CoV-2 RNA Shedding and its Temporal Association to IgG Seropositivity. medRxiv. 2020:2020.06.02.20120774.
6. Bai HX, Hsieh B, Xiong Z, Halsey K, Choi JW, Tran TML, et al. Performance of radiologists in differentiating COVID-19 from viral pneumonia on chest CT. Radiology. 2020:200823.
7. Blain H, Rolland Y, Tuaillon E, Giacosa N, Albrand M, Jaussent A, et al. Efficacy of a Test-Retest Strategy in Residents and Health Care Personnel of a Nursing Home Facing a COVID-19 Outbreak. J Am Med Dir Assoc. 2020;21(7):933-6.
8. Brill SE, Jarvis HC, Ozcan E, Burns TLP, Warraich RA, Amani LJ, et al. COVID-19: a retrospective cohort study with focus on the over-80s and hospital-onset disease. BMC Med. 2020;18(1):194.
9. Butler-Wu SM, Wald-Dickler N, Holtom P, Zangwill KM, Van TT. Under Allocation: Critical Supply Chain Hurdles Negatively Impact the Ability of Community Hospitals to Perform Repeat SARS-CoV-2 Testing. J Clin Microbiol. 2020.
10. Byrne RL, Kay GA, Kontogianni K, Brown L, Collins AM, Cuevas LE, et al. Saliva offers a sensitive, specific and non-invasive alternative to upper respiratory swabs for SARS-CoV-2 diagnosis. medRxiv. 2020.
11. Callahan C, Lee R, Lee G, Zulauf KE, Kirby JE, Arnaout R. Nasal-Swab Testing Misses Patients with Low SARS-CoV-2 Viral Loads. medRxiv. 2020.
12. Caruso D, Zerunian M, Polici M, Pucciarelli F, Polidori T, Rucci C, et al. Chest CT Features of COVID-19 in Rome, Italy. Radiology. 2020;296(2):E79-e85.
13. Cassaniti I, Novazzi F, Giardina F, Salinaro F, Sachs M, Perlini S, et al. Performance of VivaDiag COVID-19 IgM/IgG Rapid Test is inadequate for diagnosis of COVID-19 in acute patients referring to emergency room department. J Med Virol. 2020.
14. Chan JF, Yip CC, To KK, Tang TH, Wong SC, Leung KH, et al. Improved molecular diagnosis of COVID-19 by the novel, highly sensitive and specific COVID-19-RdRp/Hel real-time reverse transcription-polymerase chain reaction assay validated in vitro and with clinical specimens. Journal of clinical microbiology. 2020.

15. Chang MC, Seo WS, Park D, Hur J. Analysis of SARS-CoV-2 Screening Clinic (Including Drive-Through System) Data at a Single University Hospital in South Korea from 27 January 2020 to 31 March 2020 during the COVID-19 Outbreak. Healthcare (Basel). 2020;8(2).
16. Chau NVV, Thanh Lam V, Thanh Dung N, Yen LM, Minh NNQ, Hung LM, et al. The natural history and transmission potential of asymptomatic SARS-CoV-2 infection. Clin Infect Dis. 2020.
17. Chen CM, Jyan HW, Chien SC, Jen HH, Hsu CY, Lee PC, et al. Containing COVID-19 among 627,386 Persons Contacting with Diamond Princess Cruise Ship Passengers Disembarked in Taiwan: Big Data Analytics. J Med Internet Res. 2020.
18. Chen JH, Yip CC, Chan JF, Poon RW, To KK, Chan KH, et al. Clinical performance of the Luminex NxTAG CoV Extended Panel for SARS-CoV-2 detection in nasopharyngeal specimens of COVID-19 patients in Hong Kong. J Clin Microbiol. 2020.
19. Chen L, Deng C, Chen X, Zhang X, Chen B, Yu H, et al. Ocular manifestations and clinical characteristics of 535 cases of COVID-19 in Wuhan, China: a cross-sectional study. Acta Ophthalmol. 2020.
20. Cheng Z, Lu Y, Cao Q, Qin L, Pan Z, Yan F, et al. Clinical Features and Chest CT Manifestations of Coronavirus Disease 2019 (COVID-19) in a Single-Center Study in Shanghai, China. AJR American journal of roentgenology. 2020:1-6.
21. Clark TW, Brendish NJ, Poole S, Naidu VV, Mansbridge C, Norton N, et al. Diagnostic accuracy of the FebriDx host response point-of-care test in patients hospitalised with suspected COVID-19. J Infect. 2020.
22. Corral JE, Hoogenboom SA, Kröner PT, Vazquez-Roque MI, Picco MF, Farraye FA, et al. COVID-19 polymerase chain reaction testing before endoscopy: an economic analysis. Gastrointest Endosc. 2020.
23. Craney AR, Velu PD, Satlin MJ, Fauntleroy KA, Callan K, Robertson A, et al. Comparison of Two High-Throughput Reverse Transcription-PCR Systems for the Detection of Severe Acute Respiratory Syndrome Coronavirus 2. J Clin Microbiol. 2020;58(8).
24. Dai WC, Zhang HW, Yu J, Xu HJ, Chen H, Luo SP, et al. CT Imaging and Differential Diagnosis of COVID-19. Canadian Association of Radiologists journal = Journal l'Association canadienne des radiologistes. 2020;71(2):195-200.
25. Schwartz DA. An Analysis of 38 Pregnant Women with COVID-19, Their Newborn Infants, and Maternal-Fetal Transmission of SARS-CoV-2: Maternal Coronavirus Infections and Pregnancy Outcomes. Archives of pathology & laboratory medicine. 2020.
26. López de la Iglesia J, Fernández-Villa T, Rivero A, Carvajal A, Bay Simon E, Martínez Martínez M, et al. Predictive factors of COVID-19 in patients with negative RT-qPCR. Semergen. 2020.
27. De Smet R, Mellaerts B, Vandewinckele H, Lybeert P, Frans E, Ombelet S, et al. Frailty and Mortality in Hospitalized Older Adults With COVID-19: Retrospective Observational Study. J Am Med Dir Assoc. 2020;21(7):928-32.e1.
28. Debray MP, Tarabay H, Males L, Chalhoub N, Mahdjoub E, Pavlovsky T, et al. Observer agreement and clinical significance of chest CT reporting in patients suspected of COVID-19. medRxiv. 2020.
29. Desmet T, De Paepe P, Boelens J, Coorevits L, Padalko E, Vandendriessche S, et al. Combined oropharyngeal/nasal swab is equivalent to nasopharyngeal sampling for SARS-CoV-2 diagnostic PCR. medRxiv. 2020.
30. Dharavath B, Yadav N, Desai S, Sunder R, Mishra R, Ketkar M, et al. A one-step, one-tube real-time RT-PCR based assay with an automated analysis for detection of SARS-CoV-2. Heliyon. 2020;6(7):e04405.
31. Fernandez-Rivas G, Quirant-Sanchez B, Gonzalez V, Dolade M, Martinez-Caceres E, Pina M, et al. Seroprevalence of SARS-CoV-2 IgG Specific Antibodies among Healthcare Workers in the Northern Metropolitan Area of Barcelona, Spain, after the first pandemic wave. medRxiv. 2020.
32. Freire-Paspuel B, Vega-Marino P, Velez A, Cruz M, Garcia Bereguiain MA. High sensitivity CDC EUA SARS-CoV-2 kit-based End Point-PCR assay. medRxiv. 2020.
33. Freire-Paspuel B, Vega-Marino PA, Velez A, Cruz M, Garcia Bereguiain MA. Sample pooling on triplets to speed up SARS-CoV-2 diagnosis using CDC FDA EUA RT-qPCR kit. medRxiv. 2020.
34. Gezer NS, Ergan B, Barış MM, Appak Ö, Sayıner AA, Balcı P, et al. COVID-19 S: A new proposal for diagnosis and structured reporting of COVID-19 on computed tomography imaging. Diagn Interv Radiol. 2020.
35. Ghinai I, McPherson TD, Hunter JC, Kirking HL, Christiansen D, Joshi K, et al. First known person-to-person transmission of severe acute respiratory syndrome coronavirus 2 (SARS-CoV-2) in the USA. Lancet. 2020;395(10230):1137-44.
36. Greene DN, Dickerson JA, Greninger AL, Schmidt RL. When to re-test: An examination of repeat COVID PCR patterns in an ambulatory population. J Clin Microbiol. 2020.

37. Guan WJ, Ni ZY, Hu Y, Liang WH, Ou CQ, He JX, et al. Clinical Characteristics of Coronavirus Disease 2019 in China. N Engl J Med. 2020.
38. Guo L, Ren L, Yang S, Xiao M, Chang, Yang F, et al. Profiling Early Humoral Response to Diagnose Novel Coronavirus Disease (COVID-19).
39. Han H, Luo Q, Mo F, Long L, Zheng W. SARS-CoV-2 RNA more readily detected in induced sputum than in throat swabs of convalescent COVID-19 patients. The Lancet Infectious Diseases.
40. Hirotsu Y, Maejima M, Shibusawa M, Nagakubo Y, Hosaka K, Amemiya K, et al. Pooling RT-PCR test of SARS-CoV-2 for large cohort of 'healthy' and infection-suspected patients: A prospective and consecutive study on 1,000 individuals. medRxiv. 2020.
41. Hu S, Li Z, Chen X, Liang CH. Computed tomography manifestations in super early stage 2019 novel coronavirus pneumonia. Acta Radiol. 2020:2.84185E+14.
42. Hu Z, Song C, Xu C, Jin G, Chen Y, Xu X, et al. Clinical characteristics of 24 asymptomatic infections with COVID-19 screened among close contacts in Nanjing, China. Sci China Life Sci. 2020;63(5):706-11.
43. Hua CZ, Miao ZP, Zheng JS, Huang Q, Sun QF, Lu HP, et al. Epidemiological features and viral shedding in children with SARS-CoV-2 infection. J Med Virol. 2020.
44. Huang WH, Teng LC, Yeh TK, Chen YJ, Lo WJ, Wu MJ, et al. 2019 novel coronavirus disease (COVID-19) in Taiwan: Reports of two cases from Wuhan, China. Journal of microbiology, immunology, and infection = Wei mian yu gan ran za zhi. 2020.
45. Huang C, Wang Y, Li X, Ren L, Zhao J, Hu Y, et al. Clinical features of patients infected with 2019 novel coronavirus in Wuhan, China. The Lancet. 2020;395(10223):497-506.
46. Nagura-Ikeda M, Imai K, Tabata S, Miyoshi K, Murahara N, Mizuno T, et al. Clinical evaluation of self-collected saliva by RT-qPCR, direct RT-qPCR, RT-LAMP, and a rapid antigen test to diagnose COVID-19. J Clin Microbiol. 2020.
47. Israeli O, Beth-Din A, Paran N, Stein D, Lazar S, Weiss S, et al. Evaluating the efficacy of RT-qPCR SARS-CoV-2 direct approaches in comparison to RNA extraction. bioRxiv. 2020.
48. Rodrigues JCL, Hare SS, Edey A, Devaraj A, Jacob J, Johnstone A, et al. An update on COVID-19 for the radiologist - A British society of Thoracic Imaging statement. Clinical radiology. 2020;75(5):323-5.
49. Kanne JP, Little BP, Chung JH, Elicker BM, Ketai LH. Essentials for Radiologists on COVID-19: An Update-Radiology Scientific Expert Panel. Radiology. 2020:200527.
50. Ai JW, Zhang Y, Zhang HC, Xu T, Zhang WH. Era of molecular diagnosis for pathogen identification of unexplained pneumonia, lessons to be learned. Emerging microbes & infections. 2020;9(1):597-600.
51. Kinloch NN, Ritchie G, Brumme CJ, Dong W, Dong W, Lawson T, et al. Suboptimal biological sampling as a probable cause of false-negative COVID-19 diagnostic test results. J Infect Dis. 2020.
52. Lagier JC, Colson P, Tissot Dupont H, Salomon J, Doudier B, Aubry C, et al. Testing the repatriated for SARS-Cov2: Should laboratory-based quarantine replace traditional quarantine? Travel Med Infect Dis. 2020:101624.
53. Leber W, Lammel O, Redlberger-Fritz M, Mustafa-Korninger ME, Stiasny K, Glehr RC, et al. RT-PCR testing to detect a COVID-19 outbreak in Austria: rapid, accurate and early diagnosis in primary care (The REAP study). medRxiv. 2020.
54. LeBlanc JJ, Gubbay JB, Li Y, Needle R, Arneson SR, Marcino D, et al. Real-time PCR-based SARS-CoV-2 detection in Canadian Laboratories. J Clin Virol. 2020:104433.
55. Leung EC, Chow VC, Lee MK, Lai RW. Deep throat saliva as an alternative diagnostic specimen type for the detection of SARS-CoV-2. J Med Virol. 2020.
56. Li D, Zhang J, Li J. Primer design for quantitative real-time PCR for the emerging Coronavirus SARS-CoV-2. Theranostics. 2020;10(16):7150-62.
57. Li Y, Xia L. Coronavirus Disease 2019 (COVID-19): Role of Chest CT in Diagnosis and Management. AJR American journal of roentgenology. 2020:1-7.
58. Li Z, Yi Y, Luo X, Xiong N, Liu Y, Li S, et al. Development and Clinical Application of A Rapid IgM-IgG Combined Antibody Test for SARS-CoV-2 Infection Diagnosis. J Med Virol. 2020.
59. Lieberman JA, Pepper G, Naccache SN, Huang ML, Jerome KR, Greninger AL. Comparison of Commercially Available and Laboratory Developed Assays for in vitro Detection of SARS-CoV-2 in Clinical Laboratories. J Clin Microbiol. 2020.
60. Lin L, Jiang X, Zhang Z, Huang S, Zhang Z, Fang Z, et al. Gastrointestinal symptoms of 95 cases with SARS-CoV-2 infection. Gut. 2020.

61. Li X, Liu J, Liu Q, Yu L, Wu S, Yin X. [Optimization of a fluorescent qPCR detection for RNA of SARS-CoV-2]. Sheng wu gong cheng xue bao = Chinese journal of biotechnology. 2020;36(4):732-9.
62. Liu H, Liu F, Li J, Zhang T, Wang D, Lan W. Clinical and CT imaging features of the COVID-19 pneumonia: Focus on pregnant women and children. J Infect. 2020.
63. Liu R, Han H, Liu F, Lv Z, Wu K, Liu Y, et al. Positive rate of RT-PCR detection of SARS-CoV-2 infection in 4880 cases from one hospital in Wuhan, China, from Jan to Feb 2020. Clinica chimica acta; international journal of clinical chemistry. 2020;505:172-5.
64. Liu KC, Xu P, Lv WF, Qiu XH, Yao JL, Gu JF, et al. CT manifestations of coronavirus disease-2019: A retrospective analysis of 73 cases by disease severity. European journal of radiology. 2020;126:108941.
65. Long Q-x, Deng H-j, Chen J, Hu J, Liu B-z, Liao P, et al. Antibody responses to SARS-CoV-2 in COVID-19 patients: the perspective application of serological tests in clinical practice. medRxiv. 2020:2020.03.18.20038018.
66. Lu R, Wu X, Wan Z, Li Y, Jin X, Zhang C. A Novel Reverse Transcription Loop-Mediated Isothermal Amplification Method for Rapid Detection of SARS-CoV-2. International journal of molecular sciences. 2020;21(8).
67. Luo H, Tang QL, Shang YX, Liang SB, Yang M, Robinson N, et al. Can Chinese Medicine Be Used for Prevention of Corona Virus Disease 2019 (COVID-19)? A Review of Historical Classics, Research Evidence and Current Prevention Programs. Chinese journal of integrative medicine. 2020;26(4):243-50.
68. Matzkies LM, Leitner E, Stelzl E, Assig K, Bozic M, Siebenhofer D, et al. Lack of sensitivity of an IVD/CE-labelled kit targeting the S gene for detection of SARS-CoV-2. Clinical microbiology and infection : the official publication of the European Society of Clinical Microbiology and Infectious Diseases. 2020.
69. Medetalibeyoğlu A, Şenkal N, Çapar G, Köse M, Tükek T. Characteristics of the initial patients hospitalized for COVID-19: a single-center report. Turkish journal of medical sciences. 2020.
70. Mesoraca A, Margiotti K, Viola A, Cima A, Sparacino D, Giorlandino C. Evaluation of SARS-CoV-2 viral RNA in fecal samples. Virol J. 2020;17(1):86.
71. Miao C, Jin M, Miao L, Yang X, Huang P, Xiong H, et al. Early chest computed tomography to diagnose COVID-19 from suspected patients: A multicenter retrospective study. medRxiv. 2020:2020.03.24.20042432.
72. Kujawski SA, Wong KK, Collins JP, Epstein L, Killerby ME, Midgley CM, et al. First 12 patients with coronavirus disease 2019 (COVID-19) in the United States. medRxiv. 2020:2020.03.09.20032896.
73. Wang M, Zhou Y, Zong Z, Liang Z, Cao Y, Tang H, et al. A precision medicine approach to managing 2019 novel coronavirus pneumonia. Precision Clinical Medicine. 2020;3(1):14-21.
74. Miranda JP, Osorio J, Videla M, Angel G, Camponovo R, Henriquez-Henriquez M. Analytical and Clinical Validation for RT-qPCR detection of SARS-CoV-2 without RNA extraction. medRxiv. 2020:2020.06.24.20134783.
75. Moore NM, Li H, Schejbal D, Lindsley J, Hayden MK. Comparison of Two Commercial Molecular Tests and a Laboratory-Developed Modification of the CDC 2019-nCoV Reverse Transcriptase PCR Assay for the Detection of SARS-CoV-2. Journal of clinical microbiology. 2020;58(8).
76. Muenchhoff M, Mairhofer H, Nitschko H, Grzimek-Koschewa N, Hoffmann D, Berger A, et al. Multicentre comparison of quantitative PCR-based assays to detect SARS-CoV-2, Germany, March 2020. Euro surveillance : bulletin Europeen sur les maladies transmissibles = European communicable disease bulletin. 2020;25(24).
77. Karamese M, Ozgur D, Tarhan C, Dik Altintas S, Caliskan O, Tuna A, et al. The Prevalence of RT-PCR Positivity of SARS-CoV-2 on 10,000 Patients from Three Cities Located on the Eastern of Turkey. medRxiv. 2020:2020.06.25.20138131.
78. Norooznezhad AH, Najafi F, Riahi P, Moradinazar M, Shakiba E, Mostafaei S. Primary Symptoms, Comorbidities, and Outcomes of 431 Hospitalized Patients with Confirmative RT-PCR Results for COVID-19. The American journal of tropical medicine and hygiene. 2020.
79. Okamaoto K, Shirato K, Nao N, Saito S, Kageyama T, Hasegawa H, et al. An assessment of real-time RT-PCR kits for SARS-CoV-2 detection. Japanese journal of infectious diseases. 2020.
80. Okba NMA, Muller MA, Li W, Wang C, GeurtsvanKessel CH, Corman VM, et al. SARS-CoV-2 specific antibody responses in COVID-19 patients. medRxiv. 2020:2020.03.18.20038059.
81. Pan Y, Long L, Zhang D, Yuan T, Cui S, Yang P, et al. Potential False-Negative Nucleic Acid Testing Results for Severe Acute Respiratory Syndrome Coronavirus 2 from Thermal Inactivation of Samples with Low Viral Loads. Clinical chemistry. 2020;66(6):794-801.

82. Paramita S, Isnuwardana R, Marwan M, Alfian DI, Masjhoer DH. Clinical features of COVID-19 patients in Abdul Wahab Sjahranie Hospital, Samarinda, Indonesia. medRxiv. 2020:2020.05.27.20114348.
83. Park GS, Ku K, Baek SH, Kim SJ, Kim SI, Kim BT, et al. Development of Reverse Transcription Loop-Mediated Isothermal Amplification Assays Targeting SARS-CoV-2. The Journal of molecular diagnostics : JMD. 2020.
84. Patel MC, Chaisson LH, Borgetti S, Burdsall D, Chugh RK, Hoff CR, et al. Asymptomatic SARS-CoV-2 infection and COVID-19 mortality during an outbreak investigation in a skilled nursing facility. Clinical infectious diseases : an official publication of the Infectious Diseases Society of America. 2020.
85. Patel M, Chowdhury J, Zheng M, Abramian O, Verga S, Zhao H, et al. High Resolution CHEST CT(HRCT) Evaluation in Patients Hospitalized with COVID-19 Infection. medRxiv. 2020:2020.05.26.20114082.
86. Petrovan V, Vrajmasu V, Dimon P, Zaulet M. Evaluation of commercial qPCR kits for detection of SARS-CoV-2 in pooled samples. bioRxiv. 2020:2020.05.28.120667.
87. Pezzi L, Charrel RN, Ninove L, Nougairede A, Molle G, Coutard B, et al. Development and Evaluation of a duo SARS-CoV-2 RT-qPCR Assay Combining Two Assays Approved by the World Health Organization Targeting the Envelope and the RNA-Dependant RNA Polymerase (RdRp) Coding Regions. Viruses. 2020;12(6).
88. Pham J, Meyer S, Nguyen C, Williams A, Hunsicker M, McHardy I, et al. Performance characteristics of a high throughput automated transcription mediated amplification test for SARS-CoV-2 detection. medRxiv. 2020:2020.07.06.20143719.
89. Poggiali E, Dacrema A, Bastoni D, Tinelli V, Demichele E, Mateo Ramos P, et al. Can Lung US Help Critical Care Clinicians in the Early Diagnosis of Novel Coronavirus (COVID-19) Pneumonia? Radiology. 2020:200847.
90. Qiu YY, Wang SQ, Wang XL, Lu WX, Qiao D, Li JB, et al. [Epidemiological analysis on a family cluster of COVID-19]. Zhonghua liu xing bing xue za zhi = Zhonghua liuxingbingxue zazhi. 2020;41(4):506-9.
91. Reijns MAM, Thompson L, Acosta JC, Black HA, Sanchez-Luque FJ, Diamond A, et al. A sensitive and affordable multiplex RT-qPCR assay for SARS-CoV-2 detection. medRxiv. 2020:2020.07.14.20154005.
92. Jiang G, Ren X, Liu Y, Chen H, Liu W, Guo Z, et al. Application and optimization of RT-PCR in diagnosis of SARS-CoV-2 infection. medRxiv. 2020:2020.02.25.20027755.
93. Salehi S, Abedi A, Balakrishnan S, Gholamrezanezhad A. Coronavirus Disease 2019 (COVID-19): A Systematic Review of Imaging Findings in 919 Patients. AJR American journal of roentgenology. 2020:1-7.
94. Shirato K, Nao N, Katano H, Takayama I, Saito S, Kato F, et al. Development of Genetic Diagnostic Methods for Novel Coronavirus 2019 (nCoV-2019) in Japan. Japanese journal of infectious diseases. 2020.
95. Thomas-Ruddel D, Winning J, Dickmann P, Ouart D, Kortgen A, Janssens U, et al. [Coronavirus disease 2019 (COVID-19): update for anesthesiologists and intensivists March 2020]. Der Anaesthesist. 2020;69(4):225-35.
96. Steuwe A, Rademacher C, Valentin B, Köhler M-H, Appel E, Keitel V, et al. Dose-optimised chest computed tomography for diagnosis of Coronavirus Disease 2019 (COVID-19) – Evaluation of image quality and diagnostic impact. Journal of Radiological Protection. 2020;40(3):877-91.
97. Szymczak WA, Goldstein DY, Orner EP, Fecher RA, Yokoda RT, Skalina KA, et al. Utility of Stool PCR for the Diagnosis of COVID-19: Comparison of Two Commercial Platforms. Journal of clinical microbiology. 2020:JCM.01369-20.
98. Takeuchi Y, Furuchi M, Kamimoto A, Honda K, Matsumura H, Kobayashi R. Saliva-based PCR tests for SARS-CoV-2 detection. J Oral Sci. 2020;62(3):350-1.
99. Thieux M, Kalenderian AC, Chabrol A, Gendt L, Giraudier E, Lelievre H, et al. Assessment of a Diagnostic Strategy Based on Chest Computed Tomography in Patients Hospitalized for COVID-19 Pneumonia: an observational study. medRxiv. 2020:2020.06.29.20140129.
100. To KK, Tsang OT, Leung WS, Tam AR, Wu TC, Lung DC, et al. Temporal profiles of viral load in posterior oropharyngeal saliva samples and serum antibody responses during infection by SARS-CoV-2: an observational cohort study. The Lancet Infectious diseases. 2020;20(5):565-74.
101. tung chen y, Marti de Gracia M, DiezTascon A, Agudo-Fernandez S, Alonso-Gonzalez R, Rodriguez Fuertes P, et al. CORRELATION BETWEEN CHEST COMPUTED TOMOGRAPHY AND LUNG ULTRASONOGRAPHY IN PATIENTS WITH CORONAVIRUS DISEASE 2019 (COVID-19). medRxiv. 2020:2020.05.08.20095117.

102. Valent F, Doimo A, Mazzilis G, Pipan C. RT-PCR tests for SARS-CoV-2 processed at a large Italian Hospital and false-negative results among confirmed COVID-19 cases. Infection control and hospital epidemiology. 2020:1-2.
103. van Kasteren PB, van der Veer B, van den Brink S, Wijsman L, de Jonge J, van den Brandt A, et al. Comparison of seven commercial RT-PCR diagnostic kits for COVID-19. Journal of clinical virology : the official publication of the Pan American Society for Clinical Virology. 2020;128:104412.
104. Vancheri SG, Savietto G, Ballati F, Maggi A, Canino C, Bortolotto C, et al. Radiographic findings in 240 patients with COVID-19 pneumonia: time-dependence after the onset of symptoms. European radiology. 2020:1-9.
105. Velu P, Craney A, Ruggiero P, Sipley J, Cong L, Hissong E, et al. Rapid implementation of SARS-CoV-2 emergency use authorization RT-PCR testing and experience at an academic medical institution. medRxiv. 2020:2020.06.05.20109637.
106. Visseaux B, Le Hingrat Q, Collin G, Ferré V, Storto A, Ichou H, et al. Evaluation of the RealStar® SARS-CoV-2 RT-PCR kit RUO performances and limit of detection. Journal of clinical virology : the official publication of the Pan American Society for Clinical Virology. 2020;129:104520.
107. Vlek ALM, Wesselius TS, Achterberg R, Thijsen SFT. Combined throat/nasal swab sampling for SARS-CoV-2 is equivalent to nasopharyngeal sampling. European journal of clinical microbiology & infectious diseases : official publication of the European Society of Clinical Microbiology. 2020:1-3.
108. Wang J, Feng H, Zhang S, Ni Z, Ni L, Chen Y, et al. SARS-CoV-2 RNA detection of hospital isolation wards hygiene monitoring during the Coronavirus Disease 2019 outbreak in a Chinese hospital. International journal of infectious diseases : IJID : official publication of the International Society for Infectious Diseases. 2020;94:103-6.
109. Wang M, Wu Q, Xu W, Qiao B, Wang J, Zheng H, et al. Clinical diagnosis of 8274 samples with 2019-novel coronavirus in Wuhan. medRxiv. 2020:2020.02.12.20022327.
110. Wang X, Tan L, Wang X, Liu W, Lu Y, Cheng L, et al. Comparison of nasopharyngeal and oropharyngeal swabs for SARS-CoV-2 detection in 353 patients received tests with both specimens simultaneously. International journal of infectious diseases : IJID : official publication of the International Society for Infectious Diseases. 2020;94:107-9.
111. Wang Z, Ma W, Zheng X, Wu G, Zhang R. Household transmission of SARS-CoV-2. The Journal of infection. 2020.
112. Wang Y, Dong C, Hu Y, Li C, Ren Q, Zhang X, et al. Temporal Changes of CT Findings in 90 Patients with COVID-19 Pneumonia: A Longitudinal Study. Radiology. 2020:200843.
113. Wei M, Yuan J, Liu Y, Fu T, Yu X, Zhang ZJ. Novel Coronavirus Infection in Hospitalized Infants Under 1 Year of Age in China. Jama. 2020;323(13):1313-4.
114. Wirden M, Feghoul L, Bertine M, Nere M-L, Le Hingrat Q, Abdi B, et al. Multicenter comparison of the Cobas 6800 system with the RealStar RT-PCR kit for the detection of SARS-CoV-2. bioRxiv. 2020:2020.06.29.179184.
115. Won J, Lee S, Park M, Kim TY, Park MG, Choi BY, et al. Development of a Laboratory-safe and Low-cost Detection Protocol for SARS-CoV-2 of the Coronavirus Disease 2019 (COVID-19). Experimental neurobiology. 2020.
116. Wu J, Wu X, Zeng W, Guo D, Fang Z, Chen L, et al. Chest CT Findings in Patients With Coronavirus Disease 2019 and Its Relationship With Clinical Features. Investigative radiology. 2020;55(5):257-61.
117. Wu J, Liu J, Li S, Peng Z, Xiao Z, Wang X, et al. Detection and analysis of nucleic acid in various biological samples of COVID-19 patients. Travel medicine and infectious disease. 2020:101673.
118. Xiong Z, Fu L, Zhou H, Liu JK, Wang AM, Huang Y, et al. [Construction and evaluation of a novel diagnosis process for 2019-Corona Virus Disease]. Zhonghua yi xue za zhi. 2020;100(0):E019.
119. Xiao AT, Tong YX, Zhang S. False-negative of RT-PCR and prolonged nucleic acid conversion in COVID-19: Rather than recurrence. Journal of medical virology. 2020.
120. Xiao AT, Tong YX, Zhang S. Profile of RT-PCR for SARS-CoV-2: a preliminary study from 56 COVID-19 patients. Clinical infectious diseases : an official publication of the Infectious Diseases Society of America. 2020.
121. Xiao WJ, Gao Q, Jin K, Gong XH, Han RB, Jiang CY, et al. [Investigation of an epidemic cluster caused by COVID rectangle19 cases in incubation period in Shanghai]. Zhonghua liu xing bing xue za zhi = Zhonghua liuxingbingxue zazhi. 2020;41(0):E033.

122. Xie C, Jiang L, Huang G, Pu H, Gong B, Lin H, et al. Comparison of different samples for 2019 novel coronavirus detection by nucleic acid amplification tests. International journal of infectious diseases : IJID : official publication of the International Society for Infectious Diseases. 2020;93:264-7.
123. Xiong Y, Sun D, Liu Y, Fan Y, Zhao L, Li X, et al. Clinical and High-Resolution CT Features of the COVID-19 Infection: Comparison of the Initial and Follow-up Changes. Investigative radiology. 2020.
124. Xu T, Chen C, Zhu Z, Cui M, Chen C, Dai H, et al. Clinical features and dynamics of viral load in imported and non-imported patients with COVID-19. International journal of infectious diseases : IJID : official publication of the International Society for Infectious Diseases. 2020;94:68-71.
125. Xu J, Wu R, Huang H, Zheng W, Ren X, Wu N, et al. Computed Tomographic Imaging of 3 Patients With Coronavirus Disease 2019 Pneumonia With Negative Virus Real-time Reverse-Transcription Polymerase Chain Reaction Test. Clinical infectious diseases : an official publication of the Infectious Diseases Society of America. 2020.
126. Yang Y, Yang M, Shen C, Wang F, Yuan J, Li J, et al. Evaluating the accuracy of different respiratory specimens in the laboratory diagnosis and monitoring the viral shedding of 2019-nCoV infections. medRxiv. 2020:2020.02.11.20021493.
127. Ye Z, Zhang Y, Wang Y, Huang Z, Song B. Chest CT manifestations of new coronavirus disease 2019 (COVID-19): a pictorial review. European radiology. 2020.
128. Yoon SH, Lee KH, Kim JY, Lee YK, Ko H, Kim KH, et al. Chest Radiographic and CT Findings of the 2019 Novel Coronavirus Disease (COVID-19): Analysis of Nine Patients Treated in Korea. Korean journal of radiology. 2020;21(4):494-500.
129. Yu F, Yan L, Wang N, Yang S, Wang L, Tang Y, et al. Quantitative Detection and Viral Load Analysis of SARS-CoV-2 in Infected Patients. Clinical infectious diseases : an official publication of the Infectious Diseases Society of America. 2020;71(15):793-8.
130. Li YY, Wang WN, Lei Y, Zhang B, Yang J, Hu JW, et al. [Comparison of the clinical characteristics between RNA positive and negative patients clinically diagnosed with 2019 novel coronavirus pneumonia]. Zhonghua jie he he hu xi za zhi = Zhonghua jiehe he huxi zazhi = Chinese journal of tuberculosis and respiratory diseases. 2020;43(0):E023.
131. Xiong Z, Fu L, Zhou H, Liu JK, Wang AM, Huang Y, et al. [Construction and evaluation of a novel diagnosis pathway for 2019-Corona Virus Disease]. Zhonghua Yi Xue Za Zhi. 2020;100(16):1223-9.
132. Zhang J, Wang S, Xue Y. Fecal specimen diagnosis 2019 novel coronavirus-infected pneumonia. J Med Virol. 2020.
133. Zhao J, Yuan Q, Wang H, Liu W, Liao X, Su Y, et al. Antibody responses to SARS-CoV-2 in patients of novel coronavirus disease 2019. medRxiv. 2020:2020.03.02.20030189.
134. Zhao D, Yao F, Wang L, Zheng L, Gao Y, Ye J, et al. A comparative study on the clinical features of COVID-19 pneumonia to other pneumonias. Clinical infectious diseases : an official publication of the Infectious Diseases Society of America. 2020.
135. Zhen W, Manji R, Smith E, Berry GJ. Comparison of Four Molecular In Vitro Diagnostic Assays for the Detection of SARS-CoV-2 in Nasopharyngeal Specimens. Journal of clinical microbiology. 2020.
136. Zheng KI, Gao F, Wang XB, Sun QF, Pan KH, Wang TY, et al. Obesity as a risk factor for greater severity of COVID-19 in patients with metabolic associated fatty liver disease. Metabolism: clinical and experimental. 2020:154244.
137. Wang Z, Yang B, Li Q, Wen L, Zhang R. Clinical Features of 69 Cases with Coronavirus Disease 2019 in Wuhan, China. Clinical Infectious Diseases. 2020.
