# Supplementary material for: False-negative results of initial RT-PCR assays for COVID-19: A systematic review
Source: PLoS One. 2020 Dec 10;15(12):e0242958. doi: 10.1371/journal.pone.0242958 (PMC7728293; doi:10.1371/journal.pone.0242958)
Supplement: S4 File — (PDF) [file pone.0242958.s004.pdf]

S4 File. JBI case series tool- quality assessment of included studies

Author	1	2	3	4	5	6	7	8a	8b	9	10	11a	11b
	Clear criteria for inclusion	SARS-CoV-2 measurement reliable way for all participants	Were valid methods used for identification of the condition for all	Consecutive inclusion of participants	Complete inclusion of participants	Reporting of the demographics of participants	Reporting of clinical information	Were the outcomes or follow up results of cases clearly reported?	Follow/up adequate	Reporting of the site/clinic demographics	Numerator and denominator available	Study population adequate sample of the source population	Study population adequate sample of the target population
Ai T 2020	Yes	Yes	Yes	Unclear	Unclear	Yes	Yes	Yes	Yes	Yes	Yes	Yes	No
Albert 2020	Yes	Yes	Yes	Unclear	Yes	No	Yes	Yes	Yes	Yes	Yes	Yes	Yes
Bernheim A 2020	Yes	Yes	Yes	Unclear	Yes	Yes	Yes	Yes	Yes	Yes	Yes	Yes	No
Besutti 2020	Yes	Yes	Yes	Yes	Yes	Yes	Yes	Yes	Yes	Yes	Yes	Yes	No
Chen D 2020	Yes	Yes	Yes	Unclear	Unclear	Yes	Yes	Yes	Yes	Yes	Yes	Yes	No
Chen HJ 2020	Yes	Yes	Yes	Unclear	Yes	Yes	Yes	Unclear	Yes	Yes	Yes	Yes	No
Chen ZH 2020	Yes	Yes	Yes	Yes	Yes	Yes	Yes	Yes	Yes	Yes	Yes	Yes	No
Cinkooglu 2020	Yes	Yes	Yes	Yes	Yes	Yes	Yes	Yes	Yes	Yes	Yes	Yes	No
Dai H 2020 2020	Yes	Yes	Yes	Yes	Yes	Yes	Yes	Yes	Unclear	Yes	Yes	Yes	No
Duan X 2020	Yes	Yes	Yes	Yes	Yes	Yes	Yes	Yes	Yes	Yes	Yes	Yes	No
Fang Y 2020	Yes	Yes	Yes	Yes	Yes	Yes	Yes	Yes	Yes	Yes	Yes	Yes	No
Fechner 2020	Yes	Yes	Yes	Yes	Unclear	No	Yes	Yes	Yes	Yes	Yes	Yes	No
Gietema 2020	Yes	Yes	Yes	Yes	Yes	Yes	Yes	Yes	Yes	Yes	Yes	Yes	No
He JL 2020	Yes	Yes	Yes	Yes	Yes	Yes	Yes	Yes	Yes	Yes	Yes	Yes	No
Lan 2020	Yes	Yes	Yes	Yes	Yes	Yes	Yes	Yes	Yes	Yes	Yes	Yes	No
Lee TH 2020	Yes	Yes	Yes	Yes	Yes	No	Yes	Yes	Yes	Yes	Yes	Yes	Yes
Li Y 2020	Yes	Yes	Yes	Yes	Yes	Yes	Yes	Yes	Yes	Yes	Yes	Yes	No
Long C	Yes	Yes	Yes	No	No	Yes	Yes	Yes	Yes	Yes	Yes	Yes	No
Long DR 2020	Yes	Yes	Yes	Yes	Yes	Yes	Yes	Yes	Yes	Yes	Yes	Yes	No
Ma H 2020	Yes	Yes	Yes	Yes	Yes	Yes	Yes	Yes	Unclear	Yes	Yes	Yes	No
Richardson 2020	Yes	Yes	Yes	Yes	Unclear	Yes	Yes	Yes	Yes	Yes	Yes	Yes	Yes
Shen N 2020	Yes	Yes	Yes	Yes	Yes	Yes	Yes	Yes	Yes	Yes	Yes	Yes	No
Wang P 2020	Yes	Yes	Yes	Yes	Yes	Yes	Yes	Yes	Yes	Yes	Yes	Yes	No
Wen Z 2020	Yes	Yes	Yes	Unclear	Yes	Yes	Yes	Yes	Unclear	Yes	Yes	Yes	No
Wong HYF 2020	Yes	Yes	Yes	Unclear	Yes	Yes	Yes	Yes	Yes	Yes	Yes	Yes	No
Wu J 2020	Yes	Yes	Yes	Yes	Yes	Yes	Yes	Yes	Yes	Yes	Yes	Yes	Yes
Xie X 2020	Yes	Yes	Yes	Unclear	Yes	No	Yes	Yes	Yes	Yes	Yes	Yes	No
Young BE 2020	Yes	Yes	Yes	Yes	Yes	Yes	Yes	Yes	Yes	Yes	Yes	Yes	No
Zhang H 2020	Yes	Yes	Yes	Yes	Yes	Yes	Yes	Yes	Yes	Yes	Yes	Yes	No
Zhang JJ 2020	Yes	Yes	Yes	Unclear	Yes	Yes	Yes	Yes	Yes	Yes	Yes	Yes	No
Zhao JJ	Yes	Yes	Yes	Unclear	Yes	Yes	Yes	Yes	Yes	Yes	Yes	Yes	No
Zhifeng J 2020	Yes	Yes	Yes	Unclear	Yes	Yes	Yes	Yes	Yes	Yes	Yes	Yes	No
Zhou H 2020	Yes	Yes	Yes	Yes	Yes	Yes	No	Yes	Yes	Yes	Yes	Yes	No
Zhou S 2020b	Yes	Yes	Yes	Unclear	Unclear	Yes	Yes	Yes	Yes	Yes	Yes	Yes	No
